# Hsp70 and Its Molecular Role in Nervous System Diseases

**DOI:** 10.1155/2011/618127

**Published:** 2011-02-24

**Authors:** Giuseppina Turturici, Gabriella Sconzo, Fabiana Geraci

**Affiliations:** Department of Cellular and Developmental Biology, University of Palermo, Viale delle Scienze, 90128 Palermo, Italy

## Abstract

Heat shock proteins (HSPs) are induced in response to many injuries including stroke, neurodegenerative disease, epilepsy, and trauma. The overexpression of one HSP in particular, Hsp70, serves a protective role in several different models of nervous system injury, but has also been linked to a deleterious role in some diseases. Hsp70 functions as a chaperone and protects neurons from protein aggregation and toxicity (Parkinson disease, Alzheimer disease, polyglutamine diseases, and amyotrophic lateral sclerosis), protects cells from apoptosis (Parkinson disease), is a stress marker (temporal lobe epilepsy), protects cells from inflammation (cerebral ischemic injury), has an adjuvant role in antigen presentation and is involved in the immune response in autoimmune disease (multiple sclerosis). The worldwide incidence of neurodegenerative diseases is high. As neurodegenerative diseases disproportionately affect older individuals, disease-related morbidity has increased along with the general increase in longevity. An understanding of the underlying mechanisms that lead to neurodegeneration is key to identifying methods of prevention and treatment. Investigators have observed protective effects of HSPs induced by preconditioning, overexpression, or drugs in a variety of models of brain disease. Experimental data suggest that manipulation of the cellular stress response may offer strategies to protect the brain during progression of neurodegenerative disease.

## 1. Introduction to Heat Shock Proteins

In vitro protein folding is a self-sufficient process as folding information are intrinsic to the polypeptide primary sequence [1]. On the contrary, folding in vivo is a biological problem for many reasons. During translation, since all the interacting residues are not yet present prior to chain termination, it is not possible for the new polypeptide chain to form all the amino acid contacts that determine protein's native structures. Additionally, the hydrophobic stretches that are normally hidden inside the three-dimensional structure of a correctly folded protein are not shielded from the environment and increase the tendency to form nonnative contacts. Another impediment to correct protein folding is the highly crowded nature of the extracellular milieu. In fact, high-protein concentration dramatically increase intermolecular association constants for unfolded polypeptides. This process is therefore assisted by molecular chaperones. Molecular chaperones are proteins which recognize and selectively bind nonnative proteins to form stable complexes [2]. They play an essential role in protein folding by preventing misfolding and aggregation of folding intermediates [3–5], and keep proteins on the productive folding pathway. However, they only transiently interact with their substrate protein and are not present in the final folded product. 

Molecular chaperones comprise several highly conserved families of unrelated proteins and many of them are ubiquitous and belong to the HSP family.

HSPs are molecular chaperones which assist in the proper folding of newly synthesized proteins as well as those subject to stress-induced denaturation. HSPs also exhibit a variety of cytoprotective functions [6, 7]. In addition to their role as chaperones, HSPs inhibit the apoptosis cascade [8]. 

In the nervous system, HSPs are induced in a variety of pathological states, including cerebral ischemia, neurodegenerative disease, epilepsy, and trauma [9]. Their expression has been detected in multiple cell types, including neurons, glia, and endothelial cells [10]. HSPs also exist as extracellular proteins, released both through physiological secretory mechanisms and during necrotic cell death. HSPs in the extracellular milieu can increase stress resistance as a consequence of binding to stress-sensitive recipient cells such as neurons. HSPs can also signal danger to inflammatory cells and aid in immunosurveillance by transporting intracellular peptides to immune cells [11]. 

HSPs are classified into different families on the basis of molecular mass: Hsp100, Hsp90, Hsp70, Hsp60, Hsp40, and the small Hsp families. One of the most conserved is the Hsp70 family [12]. Almost all HSPs have a constitutively expressed member that plays a housekeeping role, and a stress-induced member that plays a crucial role in recovery after cellular stress. The feature common to both constitutive and inducible and HSPs is that they bind solvent-exposed hydrophobic segments of nonnative polypeptides permitting folding, transport, and assembly of the polypeptide through a cycle of binding and release [13–15].

The transcription factor responsible for HSP transcriptional activation is the heat shock transcription factor 1 (HSF1) [16–19]. According to the chaperone-based model, HSF1 in unstressed cells is maintained in an inactive complex with Hsp90, Hsp40, and Hsp70. When HSP levels are required in response to cellular stress, HSF1 is released from the complex and migrates to the nucleus. The active homotrimeric, hyperphosphorylated HSF1 binds heat shock elements (HSEs) in the promoter of HSP genes, leading to their upregulation [17, 19].

## 2. The Hsp70 Family

In contrast to other HSPs (e.g., Hsp90), Hsp70 proteins were found in almost the intracellular compartments. In humans, the Hsp70 multigene family includes the cytosolic and nuclear localized Hsc70 and Hsp70, endoplasmic reticulum localized Grp78, and mithochondrial MtHsp75. Hsc70, Grp78 and MtHsp75 are abundantly expressed during normal growth condition. In contrast, Hsp70 levels are growth regulated [20, 21] and induced in response to a variety of stressful stimuli in all living organisms (e.g., hyperthermia, oxidative stress, heavy metals, amino acid analogs, and mechanical stress). This protein and its constitutive form (Hsc70) are involved in different chaperoning processes, such as refolding of misfolded or aggregated proteins, preventing protein aggregation, folding and assembly of nascent polypeptides, and promoting the ubiquitination and degradation of misfolded proteins. They are also involved in protein translocation through the intracellular membrane and interactions with signal transduction proteins [22–24]. Chaperones of the Hsp70 family act by holding nascent and newly synthesized chains in a state competent for folding upon release into the medium (i.e., they are holders and not folders). The Hsp70 preferentially bind unfolded or partially folded proteins via an interaction between the chaperone and an extended polypeptide segment with a net hydrophobic character, and do not bind normal active proteins, with the exceptions of clathrin and *σ* ^32^ [25, 26].

The role of Hsp70 in the folding of nonnative proteins can be divided into three related activities: prevention of aggregation, promotion of folding to the native state, and solubilization and refolding of aggregated proteins [27].

## 3. Structural Features of Hsp70

**Figure 1:**
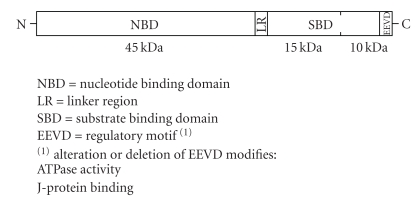
Domain structure of Hsp70.

Hsp70 and its homologs are composed of two major functional domains whose cooperation is needed for protein folding. They have an N-terminal nucleotide-binding domain (NBD) of 45 kDa, with a weak ATPase activity which can be stimulated by binding to unfolded proteins and synthetic peptides [28], and a C-terminal substrate-binding domain (SBD) of ca. 25 kDa, which is further subdivided into a *β*-sandwich subdomain of 15 kDa and a C-terminal *α*-helical subdomain. Hsp70 requires specific monovalent and divalent metal ions (K^+^ and Mg^2+^) for ATP binding and hydrolysis [29]. The NBD is structurally similar to actin and hexokinase, and it consists of four smaller domains forming two lobes with a deep cleft within which the MgATP and the MgADP bind [30]. The NBD and the SBD are connected by a short linker [31] (Figure 1). Crystal structure suggests that the *α*-helical subdomain of the SBD acts as a lid that can adopt two different states, open and closed [27, 32]. Polypeptides bind to the *β*-sandwich in an extended conformation, whereas the lid has no direct contact with the substrate. In addition, deletion mutagenesis studies have demonstrated that the C-terminal EEVD sequence motif plays an important role in cochaperone binding.

**Figure 2:**
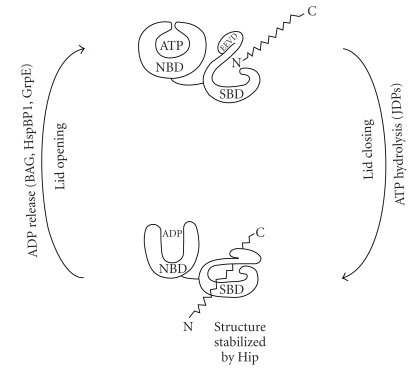
Schematic of ATP hydrolysis and the role of cochaperones.

Hsp70 adopts three different conformations, one in the absence of nucleotide, one with ADP bound, and one with ATP bound. Many of the functions of Hsp70 depend on crosstalk between the SBD and NBD, and ATP influences substrate binding. In particular, ATP binding increases the on- and off-rates of peptide binding in the adjacent SBD. Subsequently, nucleotide hydrolysis to ADP closes the lid and enhances substrate affinity [33]. Thus, in the ATP-bound state peptides can easily bind and dissociate (open lid), while in the ADP-bound state the complex with the peptide is more stable (closed lid) (Figure 2). This is a two-way communication, as interactions between SBD and its substrates increase the rate of ATP hydrolysis.

## 4. Hsp70s and Cochaperones

Since Hsp70 plays many roles within the same cellular compartment, regulation of substrate binding and/or release is essential. Hsp70 nucleotide turnover in vivo is regulated by cochaperones. In particular, Hsp40 represents a large protein family that stimulates ATP hydrolysis through a J domain (Figure 2). According to their domain composition, the members of J proteins have been subdivided into three classes based on their similarity to *E. coli* DNAJ, the prototype for all the J domain containing proteins (JDPs). Hsp70 family members often colocalize with multiple members of the Hsp40 family, which have specialized functions. Members of Hsp40 regulate substrate-Hsp70 complex formation via three mechanisms. Hsp40 proteins have unique classes of polypeptide-binding domains (PPDs) that are responsible for the selective binding of client proteins to Hsp70 [34–36]. It is reasonable to think that under in vivo conditions, Hsp70 is in the ATP-bound form, that is, in the open state. In this condition a stable binding with substrates is not possible, and experimental data suggest that J proteins first bind the substrate. This complex binds Hsp70-ATP and partially transfers the polypeptide to Hsp70. J proteins also stabilize Hsp70-polypeptides complexes by driving the conversion from the Hsp70-ATP to the Hsp70-ADP, which binds protein substrate tightly [37–39]. This Hsp40 effect may be particularly important for the Hsp70 binding of extended sequences not containing hydrophobic acid residues, such as PolyQ [40]. Finally, different Hsp40 proteins are localized to different sites within the same cellular compartment, enabling unique client binding at these sites [41–43]. On the other hand, the ADP-bound (closed lid) state is stabilized by the cochaperone Hip (or ST13), which serves to increase the half-life of substrate complexes [44] (Figure 2).

To complete the ATPase cycle, a distinct class of cochaperones, the nucleotide exchange factors (NEFs), catalyze the release of ADP. The major NEF families include the GrpE-like family, BAG family proteins, HspBP1 [45–47]. All known NEF proteins bind NBD and promote ADP release (Figure 2). By regulating ATP cycling, J-domain proteins and NEFs also control substrate binding.

All cells have evolved two mechanisms for the degradation of unfolded protein: the ubiquitin-proteasome pathway and lysosome-mediated autophagy [48]. Another group of cochaperones is represented by the tetratricopeptide repeat (TPR)-containing proteins, which bind to the EEVD sequence of the Hsp70 C-terminus. These include Hop and CHIP. The former bridges Hsp70 and Hsp90 and assists substrate transfer between the two chaperones, while the latter competes with Hop for binding the C-terminus of Hsc70 and Hsp90 [49]. CHIP also contains a U-box and acts as E3-ubiquitin ligase that ubiquitylates Hsc70 substrates, promoting their degradation by the proteasome [50–52]. Therefore, CHIP determinates whether proteins enter the productive folding pathway or the degradation pathway. In addition to catalyzing ubiquitylation of Hsp/Hsc70 and Hsp90 substrates, CHIP also ubiquitylates Hsc70 in a noncanonical manner, as it is not a degradation signal [53].

## 5. Hsp70-Mediated Protection: A Chaperone Role

Under certain pathological conditions the protein quality control machinery is not sufficient to prevent the accumulation of misfolded proteins. A common feature among various neurodegenerative diseases, including Alzheimer disease (AD), Parkinson disease (PD), amyotrophic lateral sclerosis (ALS), and the inheritable polyglutamine (PolyQ) diseases (e.g., Huntington disease (HD); spinocerebellar ataxia (SCA) type 1, 2, 3, 6, 7, and 17; spinobulbar muscular atrophy (SBMA); dentatorubral pallidoluysian atrophy (DRPLA)) is the accumulation and deposition of misfolded proteins in the brain (inside and outside neurons) and selective neuronal loss in the central nervous system (CNS) [54] (Table 1). For all of these conformational/misfolding diseases, misfolded proteins are considered a common therapeutic target [12], and many studies have focused on the neuroprotective role of HSPs. 

It has been demonstrated that neurons are particularly vulnerable to misfolded proteins, as they are postmitotic cells and are unable to dilute misfolded or aggregated proteins through cell division [12]. The aggregates are immunoreactive for ubiquitin, and most have been reported to contain molecular chaperones and components of the proteasome (see [55–60] for a summary). Molecular chaperones and components of the proteasome can also be found in aggregates formed in transgenic animal models and transfected cell cultures by various polypeptides with expanded polyQ [61–66], mutant SOD1, (familial ALS) [67], *α*-synuclein (*α*-Syn), (PD) [68], intracellular tau tangles, and extracellular plaques formed in AD [69]. The presence of these proteins suggests that protein aggregates are recognized as misfolded conformers and that cellular protein quality control mechanisms are activated in an attempt to prevent their accumulation [60]. 

These neurodegenerative disorders impact different classes of neurons. For example, in AD neuronal loss is prominent in the entorhinal cortex and hippocampus and is accompanied by neuronal loss and dementia [70]. PD is characterized by a loss of dopaminergic neurons in the substantia nigra and is accompanied by muscle rigidity, bradykinesis, and resting tremors [71]. In ALS, neuronal death involves motoneurons of the spinal cord and motor cortex, resulting in progressive muscle wasting and weakness [72]. In polyQ diseases, different regions of the brain are affected [73]. It has also been demonstrated that in rat brain these different classes of neurons show different levels of Hsc70. Spinal motoneurons, which are impacted in the low frequency disease ALS, have a very high level of Hsc70, whereas neurons in the hippocampus and entorhinal cortex, affected in the high frequency disease AD, show comparatively low levels of Hsc70. An intermediate Hsc70 levels have been found in neurons of the substantia nigra impacted affected in PD, a disease that occurs with intermediate frequency [74, 75] (Table 2). 

Although the specific proteins that aggregate in a given neurodegenerative disease are different, they all organize in amyloid-like structures with common biochemical features, such as detergent insolubility, high *β*-sheet content, and protease resistance [12, 76]. Protein deposition in *β*-sheet-rich amyloid fibrils characterizes the pathological state of these diseases, but growing evidence indicates that the toxic agents are the oligomeric and protofibrillar intermediates of the aggregation process [12, 77]. These toxic diffusible oligomers share conformational similarities, as a single monoclonal antibody can block their toxicity when applied to cultured cell models of AD, PD, and HD [78].

In recent years several studies have demonstrated that activation of the heat shock response (HSR), and in particular elevation of Hsp70 levels, has a neuroprotective effect in several models of neurodegeneration. The protective effect is believed to be related principally to its chaperone role. One example is represented by polyQ diseases. The polyQ diseases are inherited neurodegenerative diseases caused by expansion of polyQ stretches in several proteins as a result of a genetic defect characterized by a repeating trinucleotide CAG motif. CAG repeats results in expanses of glutamine, and this expansion is responsible for self-aggregation or aggregation with other proteins and for the formation of inclusion bodies in the affected neurons, leading to toxicity and cell death [79, 80]. In fact, in all cases the neuropathology of these diseases is characterized by the presence of nuclear, and sometimes extranuclear, aggregates that are immunoreactive for the mutant protein and for ubiquitin. Members of the Hsp40 and Hsp70 chaperone families have also been found to colocalize with nuclear aggregates in several polyQ diseases, both in human and mouse brains [63, 81–84]. Expression of polyQ proteins is responsible for endogenous chaperone induction in cell culture models [63, 82], while this induction is controversial for in vivo systems. For example, expression levels of Hdj1, Hdj2 (J proteins), and Hsp70 have been shown to decrease along with disease progression in the HD mouse brain, with a possible implication in the pathogenesis of the disease [66, 85]. In the same way, expression of Hsp70 in the SCA 3 Drosophila model, after an initial induction at larval stage, declines progressively with age [86]. On the contrary, induction levels of Hsp70 in response to mutant huntingtin protein with an expanded polyQ stretch differ according to subtypes of primary neuronal cultures [87]. In vitro and in vivo studies investigating the role of Hsp70 in suppressing toxicity caused by mutant polyQ proteins have been performed in cell-, yeast-, worm-, and fly-based models of polyQ disease [61, 63, 82, 88–94]. Warrick and colleagues demonstrated in a Drosophila model of polyQ disease (SCA 3) that overexpression of Hsp70 reduced the toxicity of the disease protein, but this suppression of toxicity occurred in the absence of an observable effect on protein aggregation [61]. A similar result was also observed in SCA1- and SBMA-mouse models [95, 96]. On the contrary, only modest effects on neuropathological features were observed as a consequence of Hsp70 overexpression in R6/2 HD mice [97]. Despite this result, the absence of even one allele of the hsp70.1/hsp70.3 gene significantly exacerbated the severity of the symptoms in those mice. An increase in the size of mutant protein inclusion bodies was observed, but there were no changes in the levels of fibrillar aggregates [80]. The demonstration that Hsp70, either endogenous or overexpressed, is an integral component of the in vivo physiological response to misfolding and aggregation protein disease highlights the importance of this chaperone, in view of its potential use in management of neurodegenerative disorders. In vitro and in vivo studies have demonstrated that inhibition of aggregate formation and prevention of cell toxicity is enhanced when Hsp70 and members of the Hsp40 chaperone families are overexpressed in combination and are able to work synergistically. Overexpression of these two chaperones reduced aggregate formation and apoptosis in cultured neuronal cell models of SBMA [90, 98]. In a similar manner, atomic force microscopy in aggregation experiments demonstrated that the huntingtin fragment with an expanded polyQ repeat assembles into spherical and annular structures, and molecular chaperones Hsp70 and Hsp40 act cooperatively in an ATP-dependent fashion to attenuate the assembly of these structures, thereby accelerating fibrillization [99]. Moreover, suppression of polyQ inclusion formation was also observed both in cell culture and a mouse HD model, after genetic expression of constitutively active mutants of HSF1, responsible for the induction of multiple molecular chaperone [100, 101].

A similar neuroprotective role for Hsp70 was observed in PD. PD is a neurodegenerative, multifactorial movement disorder affecting about 3% of the population over 65 years old, and is second only to AD as the most common and debilitating age-associated human neurodegenerative disorder [102]. PD is characterized mainly by progressive and selective loss of dopaminergic neurons in the substantia nigra pars compacta, with subsequent dopamine (DA) decline in the nigrostriatal pathway, and by the presence of intracytoplasmic fibrillar *α*-Syn protein aggregates (Lewy Bodies, LB) in the remaining nigral neurons. *α*-Syn is a 140-amino acid neuronal protein probably involved in regulating cell differentiation, synaptic plasticity, and dopaminergic neurotransmission. This protein is intrinsically unfolded in aqueous solution and forms differently sized soluble prefibrillar species as well as insoluble *β*-sheet-rich fibers [103–105]. It has been demonstrated that Hsp70 overexpression reduced *α*-Syn accumulation and toxicity in both mouse and Drosophila models of PD [106, 107]. In vitro studies have also demonstrated that Hsp70 can prevent *α*-Syn fibrillar assembly [107]. In particular, in vitro aggregation experiments have demonstrated that nucleotide-free Hsp70 inhibited amyloid formation, stimulating the formation of amorphous aggregates [108, 109]. A different result was observed in the presence of physiological ATP. In fact, as initially observed in a nucleotide-free system, Hsp70 was found to inhibit *α*-Syn aggregation, but at longer time points aggregation was evident. This result was explained by Roodveldt and colleagues [110] by demonstrating that *α*-Syn mediated Hsp70 depletion in an ATP-dependent manner. 

The addition of Hip, a cochaperone which is underexpressed in PD patients [111], to Hsp70 in the presence of ATP results in the abrogation of Hsp70 depletion and the suppression of the conversion of *α*-Syn into amyloid species. Small amorphous aggregates without fibrils are instead present. These data suggest that Hip exerts a stabilization of Hsp70, which supports chaperone-mediated inhibition of amyloid formation [110]. To get better insight into the process involved in vivo and investigate the interactions of chaperones constituting Hsp70 system with *α*-Syn, Ahamad [112] used the model of DNAK/DNAJ/GrpE. Studying the whole system is more likely to obtain information on the use of chaperone machinery to inhibit in vivo *α*-Syn fibril formation. Although *α*-Syn fibrillar assembly has been demonstrated to be inhibited by Hsp70, an active refolding process mediated by Hsp70 is unlikely [113]. A hypothesis that summarizes many of the results of PD studies obtained in recent years predicts that Hsp70 solubilizes *α*-Syn and promotes the degradation of its insoluble forms, both via chaperone-mediated autophagy and the proteasome [114]. 

A protective chaperone role was also observed in models of AD. The major pathological features of AD are the extracellular accumulation of amyloid-*β* peptide (A*β*) in the senile plaque and the intracellular accumulation of abnormally phosphorylated tau protein as neurofibrillary tangles. Self-assembly of A*β* produces dimers, oligomers, unstructured aggregates, and characteristic amyloid fibrils. Of these structures, oligomers are believed to be the most neurotoxic and important in the development of disease [115]. Evans and colleagues [116] observed the interaction of HSPs with various types of A*β* structures. They used freshly prepared samples and oligomers as representatives of early stages of fibril formation, and fibrils. They found that the early stages were more susceptible than fibrils to Hsp70-mediated inhibition of protein aggregation [116]. A role in AD protection was also observed when exogenous Hsp70 was administrated to rat microglial cultures. In fact, Hsp70-activated microglia showed an increase in A*β* clearance [117]. 

A new neuroprotective role related to the chaperone function of Hsp70 may have been identified in ALS. ALS is a neurodegenerative disorder affecting upper and lower motoneurons, resulting in gradual muscle weakening and loss of motoneuron function, leading to paralysis and death of afflicted individuals [118]. Some evidence suggests a link in this pathology between HSR activation and motoneuron degeneration. Twenty percent of familial ALS is due to a mutation in the gene encoding SOD1 [119]. For this reason, transgenic mice and in vitro motoneurons expressing the mutant human SOD1 are used as models of familial ALS. Studying the heat shock response in these models has demonstrated that Hsp70 levels increase during disease progression [120]. In analogy with the other conformational/misfolding diseases, overexpression of Hsp70 reduces aggregate formation in SOD1 transfected cells. Hsp70 overexpression protected motoneurons only partially in dissociated cultures of embryonic murine spinal cord from SOD1^G93A^ mutant mice [121]. Batulan and coworkers [122] demonstrated that a more effective result is obtained with coordinate upregulation of HSPs (e.g., Hsp70 and Hsp40).

However, HSPs may also protect the nervous system by a mechanism unrelated to their chaperone function. Recent studies indicate that Hsp70 can also prevent the occurrence of apoptosis in the brain. For example, the protective effects of Hsp70 in models of PD were also due to its ability to interfere with the death cascade, protecting the integrity of dopaminergic neurons from the toxic effects of 1-methyl-4-phenyl-1,2,3,6-tetrahydropyridine (MPTP) [123, 124]. In fact, Hsp70 promotes cell survival at different levels via the regulation of apoptosis-related proteins (e.g., by interacting with p53 or AIF) [125, 126], for a recent review see [127].

## 6. Hsp70 Overexpression: A Protective or Nonprotective Role?

With the demonstration that HSP overexpression can be neuroprotective, the search for a way to pharmacologically induce the overexpression of Hsp70 and associated chaperones may lead to a promising approach for the treatment of neurodegenerative diseases [128]. In particular, there have been investigations of pharmacologically active molecules that modulate HSF1, the master stress-inducible regulator [17, 19]. To this aim, collaborative drug screens to identify therapeutic agents to prevent or treat neurodegenerative diseases have been performed by different groups, using a panel of 1040 existing drugs [129, 130]. 

In 2001 Sittler and colleagues demonstrated for the first time that geldanamycin (GA) suppresses aggregation of mutant huntingtin through the induction of molecular chaperones in cell culture [128]. GA is a naturally occurring benzoquinone ansamycin that has been shown to be active in tumor cell lines. Biochemical studies have demonstrated that GA binds specifically to Hsp90, inhibiting its function [131–133]. GA also disrupts the complex between Hsp90 and HSF1, resulting in activation of the heat stress response (HSR) in mammalian cells [134–137]. Treatment with GA, through Hsp70 and Hsp40 induction, inhibits huntingtin aggregation in a cell-culture model of HD [128]. A similar protective result was obtained in a primary culture model of familial ALS [122]. GA is also responsible for affecting *α*Syn pathology and solubility. It prevents *α*-Syn aggregation in neuroglioma-transfected cells and protects them against toxicity, if cells are pretreated before transfection [138]. The protective effects of GA have also been observed in a Drosophila model of PD [139]. In vivo results were also obtained in a mouse MPTP model of PD [140]. 

Despite all of these positive results, it is well known that GA even at low concentrations is toxic to cells, and this toxicity may limit its suitability for long-term use [141]. For this reason, more extensive investigations are currently under way with GA derivatives, such as 17-allylamino-17-demethoxygeldanamycin (17-AAG) and 17-dyimethylaminoethylamino-17-demethoxygeldanamycin (17-DMAG). 17-AAG is an analogue of GA that shows less hepatotoxicity in vivo [142]. However, controversial results have been obtained with this drug in some models. Batulan and colleagues [122] observed that 17-AAG, despite having biological efficacy in tumors at doses similar to those for GA [143] and being able to enhance androgen degradation in a mouse model of SBMA [144–146], requires high and toxic concentrations to induce HSPs synthesis in a familial model of ALS [122]. On the contrary, it has been demonstrated that 17-AAG treatment successfully suppresses neurodegeneration in a Drosophila model of SCA3 and HD, and it is the most effective agent among other HSF1-activating compounds in suppressing polyQ-related neurodegeneration in Drosophila models [79]. It was demonstrated recently that 17-AAG reduced toxicity in the SBMA model through solubilization and increased clearance of the mutant protein. This clearance is mediated by the autophagic system and has no impact on the activity of the proteasome system [147]. A similar result was obtained studying *α*-Syn clearance in cellular model. In this system 17-AAG attenuates the formation of small aggregates through induction of the autophagic pathway [148]. These results suggest that Hsp70 may contribute to *α*-Syn aggregate degradation, but is not the major player. This is in accordance with results obtained in Drosophila model where a concentration of GA that did not induce Hsp70 expression was sufficient to protect neurons against *α*-Syn toxicity [106].

The GA derivative 17-DMAG, which is more potent than 17-AAG [149, 150], is also more water soluble and can be administered orally [151], making it possibly more feasible as a long-term therapeutic agent. In the SBMA model system 17-DMAG was shown to have two major activities, preferential Hsp90 client protein degradation and HSPs induction. 17-DMAG induced the upregulation of Hsp70 and Hsp40 to a greater extent than 17-AAG [146, 152]. On the other hand, treatment with 17-DMAG shifted the AR-Hsp90 chaperone complex from a mature stabilizing form with p23 to a proteasome-targeting form with Hop [153]. A marked decrease of the mutant AR polyQ was observed even without Hsp70 induction, as detected using siRNA [146].

Another compound which is able to activate HSF1 and upregulate HSP gene expression is celastrol. Celastrol is a pentacyclic triterpenoid obtained from root of Tripterygium wikfordii Hook, a perennial vine of Celastraceae family [154]. Therapeutic studies have underscored its role in the prevention of inflammatory diseases and cancer. During a screening for inhibitors of androgen signaling, it was discovered that celastrol is also an Hsp90 inhibitor. Unlike GA and its derivatives, celastrol does not compete with ATP-binding sites. Celastrol inhibits the interaction between Hsp90 and its cochaperone cdc37 [155]. This complex is involved in the stability of the IKK signalosome and, as a consequence, regulation of NF-*κ*B, a key mediator of inflammatory gene expression. Celastrol is also responsible for HSF1 hyperphosphorylation and the induction of DNA-binding activity [156]. Hsp70 induction by celastrol has several therapeutic benefits (e.g., maintaining cellular protein control status and inhibiting inflammatory responses by reducing IKK complex activation) [154]. In neurodegenerative diseases celastrol was shown to protect against polyglutamine toxicity, both in vivo and in vitro. Its protective effects are associated with decreased numbers of cells containing aggregates as well as increased SDS-solubility of the mutant polyQ protein [157]. Celastrol is also neuroprotective in vivo in models of AD and HD. Its neuroprotective effects may be due to Hsp70 induction and prevention of Hsp70-dependent activation of nuclear factor (NF)-*κ*B and tumor necrosis factor (TNF)-*α*. This inhibition reduces proinflammatory cytokine release and astrogliosis [158]. Moreover, celastrol is neuroprotective in G93SOD1 mice (i.e., ALS model) [159] and in transgenic mice models of AD [160].

**Figure 3:**
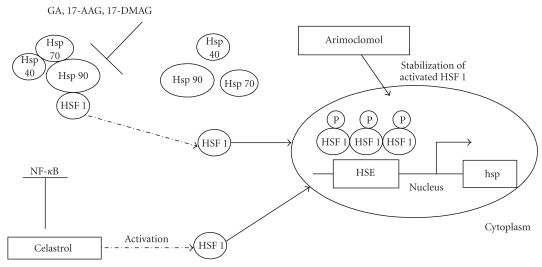
Pharmacological activation of HSF1 by small chemical activators and induction of molecular chaperones genes (hsp). 17-AAG:17-(allylamino)-17-demethoxygeldanamycin, 17-DMAG: 17-dyimethylaminoethylamino-17-demethoxygeldanamycin.

Another category of compounds which share neuroprotective activity are the coinducers of the heat shock response, that is, compounds that amplify HSP gene expression only in the presence of a concomitant stress. One such coinducer is arimoclomol, an analogue of bimoclomol, a hydroxylamine derivative [161]. These hydroxylamine derivatives have been shown to coinduce HSP expression by prolonging activation of HSF1 [162]. Kieran and colleagues showed that arimoclomol treatment significantly delays disease progression in SOD1^G93A^ mice [163]; see Figure 3. These results are summarized in Table 3.

Hsp70 overexpression, however, is not beneficial in all instances. Recently Kalmar and Greensmith [164] demonstrated that an increase in intracellular HSPs in vitro is not always beneficial for the survival of motoneurons. For example, celastrol and arimoclomol both induce Hsp70 synthesis, but they have opposite effects on motoneuron survival. In fact, whereas treatment with arimoclomol was clearly neuroprotective [163, 164], celastrol not only showed no beneficial effects on motoneurons, but actually induced caspase-mediated apoptosis [164]. The two agents, although similar in their capacity to induce Hsp70, have some important differences. In particular, as described above, arimoclomol can only function as a coinducer of the HSR [161, 162], whereas celastrol can directly induce Hsps in vitro, even in the absence of a stressor [75]. The result of this study emphasizes that caution is needed when proposing drugs that upregulate HSP levels as potential therapeutic agents for neurodegenerative disorders. 

An uncertain role for Hsp70 was also observed in an epilepsy model. A model used widely for studying the pathological changes of human temporal lobe epilepsy (TLE) is the kainic acid- (KA-) induced seizure model in rodents [165], which reproduces many of the clinical features of TLE [165–168]. KA induces Hsp70 expression in hippocampal neurons or more broadly throughout the brain, depending on the dose of KA [169, 170]. Despite the expression of Hsp70, neuroprotection was not observed during an epileptogenic state, and Hsp70 overexpression in this scenario served only as an indicator of neuronal stress in the acute phase of epilepsy [171, 172]. A different result was obtained previously by Yenari and coworkers [173]. This study showed that overexpression of Hsp70 prior to neuronal insult improves cell survival in both stroke and epilepsy models. This result demonstrated that using gene transfer for Hsp70 overexpression improved neuronal survival, although for gene therapy to have significant clinical relevance, future studies should explore whether the Hsp70 overexpression can protect neurons when administered after insult [173].

## 7. Extracellular Hsp70

HSPs exist not only as intracellular proteins, but also as extracellular proteins [11], and several reports have shown that HSPs can be released from mammalian cells [174, 175]. In recent years an extracellular role for Hsp70 has been demonstrated, and numerous functions have been attributed to it: cytokine production and release, microglial activation, induction of IL-6 and TNF-*α*, stimulation of phagocytosis, and clearance of A*β* [116, 176, 177]. 

Although the protective system based on Hsp70 exists in all tissue and organs, some cell types do not appear to express the protein. Among these are certain types of neurons [178]. For example, stress was shown to significantly increase Hsp70 mRNA expression in neurons in the cerebellum, but not in hippocampal neurons [179]. It is now well known that Hsp70 can be released from some cells and taken up by others in a biologically active form [178, 180, 181]. Although there is no evidence on how Hsp70 works extracellularly, there is evidence that Hsp70 can be internalized and imported into the cytoplasm and nucleus of many cell types to promote cell survival [176, 182–187]. 

Most neurons contain high levels of Hsc70 and low levels of Hsp70 [188]. In motoneurons both HSP forms are present, but no increase in endogenous expression is observed in the face of either insufficient trophic factors or heat shock. This apparent inability to increase Hsp70 expression may render motoneurons vulnerable to metabolic stress. Extracellular Hsp70 may, therefore, play a compensatory role after stress to promote survival, inhibit apoptosis, or both. The first observation of the stress tolerance-enhancing activity of exogenous Hsc/Hsp70 was reported by Johnson and coworkers, who showed that Hsc/Hsp70 added to the culture medium in vitro can bind to arterial smooth muscle cells and improve their resistance to nutrient-deprivation stress [182, 183]. A protective effect was also observed in cultured monocytes [184].

Extracellular Hsp70 has also been shown to enter human motoneurons [178, 189, 190]. There are several reports substantiating the protective role of exogenous Hsc/Hsp70 in the CNS. Tidwell and coworkers showed that administration of a mixture of Hsc/Hsp70 in vivo inhibits motor and sensory neuron degeneration after sciatic nerve axotomy [186]. A protective effect of exogenous recombinant human Hsp70 (rhHsp70) on motoneurons was also demonstrated by Robinson et al. (2005) [190]. They demonstrated that Hsp70 protects motoneurons deprived of trophic factors in vitro as well as those undergoing natural cell death in vivo. rhHSC70 confers protection to motoneurons subjected to the oxidative stress common in neurodegenerative diseases such as SBMA, AD, PD, and ALS [190]. Moreover, Guzhova et al. (2001) [178] demonstrated that cultured glioma cells, an in vitro model of glia-like cells, released Hsp70 in the culture medium. Furthermore, a mixture of bovine Hsc/Hsp70 was taken up by cultured neuroblastoma cells, an in vitro model of neuron-like cells. Numerous observations indicate that glial cells supply neighboring neurons with specific proteins and trophic factors, and may be a means by which motoneurons obtain Hsp70 during stressful conditions [180, 191, 192]. 

A protective role for extracellular Hsp70 was also observed in brain disease. In particular, Hsp70 has been demonstrated to be involved in protecting motoneurons against degeneration in a mouse model of ALS [193]. In fact, treatment of mice with rhHsp70 delayed symptom onset and increased lifespan. This rhHsp70 localized primarily to skeletal muscle and was not found in the CNS, suggesting a potential peripheral mode of action for the survival-promoting effect. The effect of rhHsp70 may be mediated by its action to help maintain motoneuron innervation in skeletal muscle. Furthermore, a protective effect of exogenous Hsc/Hsp70 was demonstrated in cells containing polyQ inclusions. The exogenous HSPs penetrate the cell and colocalize with inclusions. The chaperone also decreased the number of apoptotic cells [189].

Epilepsy, for which the underlying neuronal defects are distinct from those in conformational/misfolding diseases, has also been used to demonstrate an Hsp70-mediated neuroprotective effect. In two different models of epilepsy, Ekimova et al. [194] demonstrated that exogenous Hsc and Hsp70 can penetrate into brain areas (e.g., cortex, hippocampus, thalamus, hypothalamus, and pontine reticular formation) involved in the initiation and propagation of generalized tonic-clonic seizures [195–197], where it acts to attenuate the severity of chemically induced seizures [194]. This study demonstrated for the first time that exogenous Hsc and Hsp70 have anticonvulsant properties and are able to pass through the cerebrospinal fluid-brain barrier and cross the plasma membrane of neurons.

In summary, there is evidence that Hsp70 is not only an intracellular chaperone but has also extracellular functions. Many papers have demonstrated that the extracellular Hsp70/Hsc70 have protective role on neurons, and they have also a neuroprotective effect in many brain diseases (e.g., ALS, epilepsy, PolyQ). This role opens a new scenario in brain disease therapy.

## 8. Hsp70 and Immunomodulation: A Negative Role in Autoimmune Diseases

Immune activation within the CNS is a classical feature of ischemia, neurodegenerative diseases, immune-mediated disorders, infections, and trauma, and may often contribute to neuronal damage. It has been demonstrated that HSPs are able to induce the innate immune system through their interactions with cell surface receptors, leading to the expression of proinflammatory cytokines [177, 198], chemokines [199, 200], and activation of dendritic cells (DCs) [201, 202]. Hsp70 is the principal HSP implicated in the formation of the immunogenic complex [203]. In fact, a role for Hsp70 as facilitator of immune response to proteins and peptides has been demonstrated both in vivo and in vitro [204–208]. For an immune response to be activated, an antigen must be processed to lymphocytes in the context of accessory molecules expressed on the surface of antigen-presenting cells (APCs). For most T cells, these accessory molecules are represented by either class I or class II components of the major histocompatibility complex (MHC). Many reports showed that Hsp70 enhances antigen presentation through the MHCI antigen presentation pathway. In addition, Mycko and coworkers [209] demonstrated that Hsp70 is also able to promote antigen presentation by the MHC class-II-dependent pathway. It has been demonstrated for both MHC class-I- and II-dependent systems that Hsp70-associated peptides are more immunogenic than the peptides alone [209–212].

Multiple sclerosis (MS) is a chronic inflammatory CNS disease of autoimmune etiology, caused by an inappropriate immune T-cell-mediated response to CNS myelin antigens [213, 214]. In this disease, myelin antigens such as myelin basic protein (MBP), one of the most immunogenic proteins of the CNS and synthesized in the CNS only by oligodendrocytes, proteolipid protein (PLP), myelin oligodendrocyte glycoprotein (MOG), myelin-associated glycoprotein, and nonmyelin antigens such as *αβ*-crystallin, transaldolase, and CNPase, are believed to be targets of pathogenic T cells [215–222]. Once activated, these cells breach the bloodbrain barrier and migrate into the CNS, mediating the development of inflammatory foci and myelin destruction [213]. Cumulative data indicate that once damage to the CNS has occurred, sensitization to other antigens can occur, contributing to the chronic disease. 

HSPs are also believed to be permissive factors in various autoimmune diseases. In particular, anti-Hsp70 autoantibodies were found to be significantly higher in the cerebrospinal fluid of patients with multiple sclerosis (MS) than in cerebrospinal fluid from patients with motoneuron disease. Moreover, Hsp70 was found in and around MS lesions [220, 223–225], often in association with PLP [226], as well as in experimental autoimmune disease (EAE), which can be induced in rodents by myelin antigens administration (e.g., MOG, PLP, and MBP) [227, 228] and is considered a model of MS [229]. 

Myelin represents a complex multilamellar membrane, containing many myelin-specific proteins. The two major myelin proteins of the myelin sheath are PLP and MBP. PLP is an intrinsic membrane protein assembled in the endoplasmic reticulum into vesicles targeted for the cell membrane and not likely to require chaperoning by Hsc70. In contrast, MBP is synthesized on free polysomes found mostly in oligodendrocyte processes, and a possible role for Hsc70 as chaperone has been postulated [230]. It is also reasonable to hypothesize that Hsc70 should be similarly required for remyelination during the process of lesion repair. The association of Hsc70 with myelin proteins on cell membrane during this phase of the disease could function as an additional target of the immune response. Remyelination could also be impaired by a reduction in Hsc70. In fact, the Hsc70 content in autopsy tissue of MS lesions has been found 30% to 50% below that in normal brain tissue, with chronic lesions showing the lowest expression [224, 231]. This reduction could be responsible for permanent loss of myelin from the lesion [230]. On the contrary, analysis of expression and distribution of HSPs in MS lesions indicates a significant upregulation of most classes of HSPs, both within the lesion and at the lesion edge [222, 224, 226, 232]. In early active and chronic active lesions, immunoreactivity for Hsp70 was strongly positive on reactive astrocytes and some macrophages at the leading edge [230]. Hsp70 upregulation was also observed in the inflammatory lesions in the CNS of EAE animals [230]. To determine whether Hp70 overexpression is restricted to the CNS, Cwiklinska et al. 2010 [233] assessed Hsp70 expression in PBMCs from MS patients. They observed no general upregulation in these patients compared to healthy donors, but upon cell stress Hsp70 was found to be significantly overexpressed. Despite this upregulation, whether Hsp70 plays a protective or pathological role is still controversial. Hsp70 was found to be associated with MBP and PLP in CNS from MS, but not in control tissue [226]. A similar result was obtained in EAE, indicating that the demyelination process favors the physical association of HSP with myelin proteins [226]. In the bound form, Hsp70 with myelin proteins may be targeted to APC and, using an adjuvant-like mechanism, enhance an immune reaction to myelin antigens. This assumption was confirmed by the data obtained by Chen et al. [234]. They demonstrated that Hsp70 overexpression significantly enhanced the uptake of MBP by APC. Similarly, Mycko et al. [203] demonstrated that Hsp70 overexpression in vitro leads to enhanced presentation of MBP in a MHC class-II-dependent manner [203, 208, 235–239]. Hsp70 have also been shown to stimulate immune cells to produce cytokine and chemokines, which activate APC [240].

In vivo experiments also demonstrated that Hsp70 is involved in EAE resistance. In fact, hsp70.1^−/−^ mice were resistant to EAE after immunization with MOG peptide, and reduced clinical signs were also evident [203]. The results obtained by Mycko and coworkers [203] using hsp70.1^−/−^ mice demonstrated that Hsp70 is essential for the induction of the autoimmune response to the peptide MOG_35-55_. A similar result was obtained by Lund et al. [241]. They demonstrated that Hsp70 was associated with MBP peptides in normal-appearing white matter of both MS and normal human brain. They also found an adjuvant-like effect of Hsp70-associated MBP-derived peptides. Based on these results, they hypothesize that a small dose of Hsp70-MBP peptide secreted by stressed oligodendrocytes stimulated an in vivo adaptive immune response specific for the associated autoantigen. This event could be the mechanism responsible for the initiation of MS and could be responsible for the subsequent immune-mediated destruction of myelin characteristic of the disease. 

A different results was obtained by Galazka et al. [242]. They demonstrated that mouse immunization with an Hsp70 fraction associated with peptide complexes (pc) isolated from animals with EAE reduced the subsequent induction of EAE. According to this work, Hsp70 complexed with an endogenous peptide is able to regulate the immune process in a MHC class-II-dependent disease, but no results were obtained by using Hsp70-pc isolated from healthy donors. The different results between immunization with Hsp70-pc from healthy donors or Hsp70-pc from EAE donors suggests substantial differences in the peptide that binds Hsp70 in normal versus pathological CNS. As no resistance to EAE induction was obtained by immunization with a pure peptide fraction or pure Hsp70 preparation, the authors concluded that Hsp70 serves as a natural adjuvant, and that the Hsp70-pc complex is able to induce a pathway involving NK cells, inhibiting autoreactive T cells [242]. In conclusion, Hsp70 is thought to contribute to the induction and development of EAE [203], and peptides derived from inflamed CNS tissues bind to Hsp70 and inhibit EAE development [242]. Moreover, an Hsp70 protective effect was observed in celastrol-treated mice. Celastrol is responsible for Hsp70 induction and for its nuclear translocation. Furthermore, a direct interaction between NF-kB and Hsp70 was observed, leading to a decreased recruitment of inflammatory cells into the CNS [243].

## 9. Conclusions

In this paper, we have briefly focused on some of the current areas of research on molecular role of Hsp70 in nervous system diseases. Many neurodegenerative disorders are linked together by the presence and accumulation of misfolded proteins. Several works have demonstrated that Hsp70 may have a neuroprotective role in several model of neurodegeneration both in vivo and in vitro. Its beneficial effects could be due both to its chaperone role and to its ability to protect against various kinds of potentially toxic factors. These apparently positive results prompted pharmacological studies on active molecules which act on HSP modulation. However, Hsp70 protects against some but not all kinds of CNS injury and the protective effects may be related to the nature and the severity of the insults. An uncertain role on beneficial effects of Hsp70 was observed in some cases of its overexpression or in some models of brain disorders. Furthermore, HSPs are also believed to be permissive in various autoimmune diseases. In this situation either beneficial or harmful effects could be hypothesized. In particular, in MS beneficial effects could be due to mechanisms which downregulate the immune response. On the other hand, harmful effects might include the development and/or recruitment of additional antigenic targets within the lesion with the consequent amplification of the immunological response. Further studies will be required to describe the apparently contradictory roles of Hsp70 in nervous system diseases.

## Figures and Tables

**Table 1:** Neurodegenerative diseases and protein deposits.

Disease	Inclusion	Abnormal protein
Alzheimer disease	Extracellular neuritic plaque	A*β* peptide
	Cytosolic neurofibrillatory tangles	Tau
Parkinson disease	Lewy bodies	*α*-synuclein
Familial amyotrophic lateral sclerosis	Intracellular inclusions	SOD1
Huntington disease	Nuclear, cytosolic inclusion	Huntingtin
Spinocerebellar ataxia 1, 2, 3	Nuclear inclusions	Ataxin 1, 2, 3
Spinobulbar muscular atrophy	Nuclear inclusions	Androgen receptors

**Table 2:** Hsc70 and neurodegenerative diseases.

Disease	Neurons affected	Hsc70 levels	Frequency
Alzheimer disease	entorhinal cortex and hippocampus	Low	High
Parkinson disease	dopaminergic neurones in the substantia nigra pars compacta	Intermediate	Intermediate
Amyotrophic lateral sclerosis	motor neurons of the spinal cord and motor cortex	High	Low

**Table 3:** 

Drug	Neurodegenerative disease	Reference
	Cell culture model of Huntington Disease	[112]
	Mouse model of Huntington Disease	[52]
GA	Drosophila model of Parkinson Disease	[123]
	Mice MPTP (Parkinson Disease)	[124]
	Cell culture model of *α*-synuclein aggregation	[122]
	SOD^G93A^ cells (Amyotrophic Lateral Sclerosis)	[106]

	Drosophila model of PolyQ	[131]
17-AAG	Cell culture model of Huntington Disease	[135]
	Cell culture model of *α*-synuclein aggregation	[133]
	Spinobulbar Muscular Atrophy transgenic mice	[128, 129]
	SOD^G93A^ cells	[106]

17-DMAG	Mouse model of Spinobulbar Muscular Atrophy	[130, 137]

	Cell culture model of PolyQ	[142]
Celastrol	Mice MPTP (Parkinson Disease),	[143]
	Mice 3-NP (Huntington Disease)	
	SOD^G93A^ transgenic mice	[144]
	Transgenic mouse model of Alzheimer Disease	[145]

Arimoclomol	SOD1^G93A^ mice (Amyotrophic Lateral Sclerosis)	[148]
